# Chemical Characterization and Antioxidant Potential of Wild *Ganoderma* Species from Ghana

**DOI:** 10.3390/molecules22020196

**Published:** 2017-01-25

**Authors:** Mary Obodai, Deborah L. Narh Mensah, Ângela Fernandes, Nii Korley Kortei, Matilda Dzomeku, Matthew Teegarden, Steven J. Schwartz, Lillian Barros, Juanita Prempeh, Richard K. Takli, Isabel C. F. R. Ferreira

**Affiliations:** 1 CSIR-Food Research Institute, Mycology Unit, P.O. Box M20, Accra, Ghana; obodaime@yahoo.com (M.O.); lnarh@yahoo.com (D.L.N.M.); matildadela@yahoo.com (M.D.); juanita_luv06@yahoo.com (J.P.); rtakli@yahoo.com (R.K.T.); 2 Mountain Research Centre (CIMO), ESA, Polytechnic Institute of Bragança, Campus de Santa Apolónia, 1172, Bragança 5300-253, Portugal; afeitor@ipb.pt (Â.F.); lillian@ipb.pt (L.B.); 3 Department of Nutrition and Dietetics, School of Allied Health Sciences, University of Health and Allied Sciences, PMB 31, Ho, Ghana; nii_korley_1@yahoo.com; 4 Department of Food Science and Technology, The Ohio State University, Columbus, OH 43210, USA; teegarden.3@buckeyemail.osu.edu (M.T.); schwartz.177@osu.edu (S.J.S.); 5 Laboratory of Separation and Reaction Engineering-Laboratory of Catalysis and Materials (LSRE-LCM), Polytechnic Institute of Bragança, Campus de Santa Apolónia, 1134, Bragança 5301-857, Portugal

**Keywords:** *Ganoderma* species, chemical, antioxidant properties, wild mushrooms

## Abstract

The chemical characterization and antioxidant potential of twelve wild strains of *Ganoderma* sp. from Ghana, nine (LS1–LS9) of which were found growing wild simultaneously on the same dying *Delonix regia* tree, were evaluated. Parameters evaluated included the nutritional value, composition in sugars, fatty acids, phenolic and other organic compounds and some vitamins and vitamin precursors. Antioxidant potential was evaluated by investigating reducing power, radical scavenging activity and lipid peroxidation inhibition using five in vitro assays. Protein, carbohydrate, fat, ash and energy contents ranged between 15.7–24.5 g/100 g·dw, 73.31–81.90 g/100 g, 0.48–1.40 g/100 g, 0.68–2.12 g/100 g ash and 396.1–402.02 kcal/100 g, respectively. Fatty acids such as linoleic, oleic and palmitic acids were relatively abundant. Free sugars included rhamnose, fructose, mannitol, sucrose and trehalose. Total tocopherols, organic acids and phenolic compounds’ content ranged between 741–3191 µg/100 g, 77–1003 mg/100 g and 7.6–489 µg/100 g, respectively. There were variations in the β-glucans, ergosterol and vitamin D_2_ contents. The three major minerals in decreasing order were K > P > S. *Ganoderma* sp. strain AM1 showed the highest antioxidant activity. This study reveals, for the first time, chemical characteristics of *Ganoderma* spp. which grew simultaneously on the same tree.

## 1. Introduction

*Ganoderma* species are a group of very diverse polypores, which can usually not be easily distinguished based on morphological characteristics. Studies on identification of *Ganoderma* spp. using molecular techniques began recently. This has resulted in the naming of new species of the genus from Ghana [1,2,3].

*Ganoderma lucidum* is an important species in the *Ganoderma* genera abundant in numerous bioactive polysaccharides and some other molecules, consequently making it superior and more attractive among other species of medicinal mushrooms [4,5]. It is a polypore group fungus that has been used to suppress the growth rate of tumors in patients [6] and is well known in China, Japan, USA and other parts of the world as a useful source of medicine.

β-Glucans in *Ganoderma* are the major biologically active polysaccharides. The anticancer and antimetastatic activities of *Ganoderma* spp. are attributed to these polysaccharides [7]. These compounds are linked to their immunostimulating and antioxidant capacities. β-glucans are prominent among the number of fungal polysaccharides described as they contain mainly β (1→3)-glycosidic bonds and have side chains linked by β (1→6)-glycosidic bonds [8].

Fruits, whole grains, vegetables, spices, and herbs contain appreciable amounts of natural antioxidants. Mushrooms have also been reported to exhibit variable ranges of antioxidant activity, which is likely related to their major phenolic compounds despite minute quantities of ascorbic acid, carotenoids, tocopherol, etc. [9]. Phenolic acids predominate in the phenolic profile of edible mushrooms, but some species have been reported to contain varying amounts of catechin and myricetin as well [10].

There is more interest in the pharmaceutical properties than the nutritional attributes of *G. lucidum*, which is atypical compared to other cultivated mushrooms. Many *G. lucidum* products are available commercially, including powders, dietary supplements, and tea, which are produced from the mushroom’s mycelia, spores, and fruit body.

Previous work suggest that *Ganoderma* mushrooms exhibit biological activities including immunomodulating, antiallergic, antiradiation, antitumor, anti-inflammatory and antiparasitic properties [7]. Also, two triterpenes found in *Ganoderma lucidum*, ganoderic and lucidenic acid, have been shown by Kim and Kim [11] to exhibit anticancer, antiinflamatory, antihistamine and hypotensive activities.

Ergosterol is one of the primary sterols in the cell membrane of fungi, and signals for the expression of several defense genes, contributing to pathogen resistance of the organisms [12]. Nevertheless, not all fungi produce ergosterol, and the ergosterol concentration even differs within the same species depending on its physiological state. Importantly, ergosterol is the precursor to vitamin D_2_, and its conversion is facilitated by exposure to UV light [13]. When yeast and fungi are exposed to UV light, they can produce high levels of vitamin D_2_. Vitamin D_2_ therefore serves as an important available dietary source of vitamin D for vegetarians, although such persons can obtain ample vitamin D through exposure to sunlight [14].

Only a few of the thousands of mushroom species recorded worldwide can be cultivated [15]. However, research is being conducted in developing countries like Ghana on cultivation of local species [16,17]. Ghana and other countries in Africa are actively exploring the cultivation of *Ganoderma* species on several substrates. Local men and women linked to forest-related occupations like game hunting, farming, foresters, firewood collecting, palm fronds and nut gatherers have high knowledge of mushroom heritage [3]. Osarenkhoe et al. [18] deduced that wild mushrooms in the sub region are used for medicines (tentative), psychoactive effects (psychedelic or entheogenics), cosmetics (e.g., colouring of hair), flavourings, and food.

Over the years cultivation trials in Ghana of *Ganoderma* species have been carried out on agricultural wastes such as sawdust, rice straw and grasses (Juncao) with varying yields on sawdust using the plastic bag method [19]. In Ghana, the health benefits of *Ganoderma* spp. are presently based on folklore, tradition, and some documentary evidence. The objective of this study was to investigate the chemical and antioxidant properties of twelve wild *Ganoderma* species from the Greater Accra Region of Ghana with the goal of its eventual use in biotechnological applications.

## 2. Results and Discussion

### 2.1. Nutritional, Free Sugars and Beta-Glucans Composition

The nutritional attributes of mushrooms are directly linked to their chemical composition. All the analyses revealed that the *Ganoderma* spp. contained appreciable amounts of nutrients which were generally significantly different (*p* < 0.05) among the samples irrespective of the collection site (Table 1).

The protein content of the different species investigated ranged between 15.7 and 24.5 g/100 g·dw corresponding to strains AM1 (from Amrahia) and LS1 (from Legon), respectively. Despite the significance differences between them, the LS1 to LS9 samples had significantly higher protein content than the samples collected from the other locations.

Factors such as the growth substrate, pileus size, harvest time and species of mushrooms influence mushroom nutritional compositions, including protein content, and other biochemical characteristics [20,21]. Cultivated edible mushrooms can contain 3.5%–4% of their fresh weight as protein [21]. Ogbe et al. [15] reported a value of 13.3 g/100 g dry matter in *Ganoderma* spp. used in supplementing chicken feed. In another study [22], reported values of 22.6 g/100 g·dw in *G. applanatum.*

Carbohydrate content of the studied *Ganoderma* strains ranged between 73.31 and 81.90 g/100 g·dw for strains LS1 and AM1 respectively. Unlike the protein content of the strains, the samples collected from Dodowa, Haatso and Armrahia (DS1, HS111 and AM1) all contained significantly higher carbohydrate content than the LS1 to LS9 samples. Again, there were significant differences (*p* < 0.05) in the carborhydrate content of the LS1 to LS9 strains. The presently reported values are in agreement with values (63.3%) reported by Ogbe and Affiku [23]. In contrast, Usman et al. [24] reported 17.55% of carbohydrate in wild *G. lucidum* using the same analytical method.

The average fat content in mushrooms is reported to be generally low, ranging from 0.6% to 3.2%. Fat content of the *Ganoderma* spp*.* presently studied ranged between 0.48 and 1.40 g/100 g·dw and showed significant differences (*p* < 0.05) among the strains analysed. The range of fat content obtained was comparable to the 1.52% reported by Ogbe and Affiku [23]. In another study, a range of 2.8%–4.8% was reported by Takshak et al. [25] for *G. lucidum* collected throughout Haryana, India. The results of crude fat estimated were consistent (minimum 2.8%) with earlier studies by some researchers [26,27].

Ash content ranged between 0.68 and 2.12 g/100 g·dw. There were significant differences (*p* < 0.05) observed between the strains, and this could be attributed to the varying degrees of effective bioaccumulation mechanisms which make *Ganoderma* spp. take up mineral elements from the ecosystem [28]. Relatively higher value of ash content (8.4%) was reported by Ogbe and Affiku [23]. Likewise, Singh et al. [29] reported values of 8.3% and 7.8% for *G. lucidum* and *G. phillipii,* respectively, from Uttarakhand, India.

Energetic values of our samples obtained ranged from 396.1 to 402.9 kcal/100 g·dw. Again, significant differences (*p* < 0.05) were recorded among the strains irrespective of the collection site. Manzi et al. [30] emphasized that mushrooms could be classified as a dietetic food, due to their high water content and low caloric value. Ogbe and Affiku [23] reported values within the range of 1417.3 kcal/100 kg. Also Stojković et al. [31] reported values of 411 and 386 kcal/100 g·dw for *G. lucidum* from Serbia and China, respectively. Takshak et al. [25] reported lower values, ranging from 292–198 kcal/100 g·dw from different agroclimatic zones of India. These results indicate that various strains of *Ganoderma* sp. could be considered good sources of nutritional compounds.

Free sugar composition of the different *Ganoderma* spp. strains investigated are summarized in Table 1. Rhamnose, fructose, mannitol, sucrose and trehalose were the reducing sugars identified. Strain LS7 was found to contain the highest total sugars (14.1 g/100 g·dw), while strain HS1 recorded the lowest total sugar content. All strains contained all five sugars except strain LS4 which contained only two sugars (rhamnose and sucrose). There were significant differences (*p* < 0.05) observed in the total sugars and according to Hoa and Wang [32], the differences in sugar synthesis could be attributed to the variability of the range of carbon sources available for growth of mycelium. However, considering that all the LS strains grew on the same plant simultaneously although on different parts, the recorded differences in the studied nutritional parameters can better be attributed to genetic factors. Takshak et al. [25] also found fructose, mannitol and trehalose in a collection of wild *Ganoderma* spp. from India, but in much lower amounts than that found in this work using a calorimetric technique. Usman et al. [24] also identified the same free sugars (fructose, mannitol and trehalose) in a sample of *G. lucidum*. Heleno et al. [33] reported a similar profile (with the same five sugars) and total amount (10.29 g/100 g·dw) in a sample of *G. lucidum* from Portugal analyzed using the presently described methods.

Data regarding mushroom polysaccharides have been collected from different species of higher fungi and some specific polysaccharides with the identified individual sugars quantified in different mushrooms: rhamnose, xylose, fucose, arabinose, fructose, glucose, mannose, mannitol, sucrose, maltose, and trehalose [34,35].

There were appreciable levels of β-glucans observed in the samples analysed. Values ranged from 0.22% to 11.47% dw, although only traces of β-glucans were recorded in sample DS1. Strain LS7 presented the highest concentration (Table 1). The content of β-glucans in this study was found to be lower than huitlacoche mushrooms (20–120 mg/g·dw) but higher than that reported for corn (0.5–3.8 mg/g) and similar to other edible mushrooms [36]. As previously explained in the preceding paragraphs, the differences in the nutritional contents analysed among the samples could be more biotic (genetic) than abiotic (environmental).

### 2.2. Fatty Acids

Results regarding fatty acid composition, total saturated fatty acids (SFA), monounsaturated fatty acids (MUFA) and polyunsaturated fatty acids (PUFA) are shown in Table 2. Twenty-two fatty acids were quantified in *Ganoderma* strain LS6 while 21 were quantified in strain HS1. Generally, a higher occurrence of SFA over PUFA was observed, except for strains LS1, LS2, LS7 and AM1 where PUFA were dominant. It is worth noting that the LS1, LS2, LS3, LS5, LS6, LS7 and LS9 samples, which have been reported [3] to be in the same clade within the *Ganoderma lucidum* complex, generally showed significant differences in their total SFA, MUFA and PUFA. Unfortunately the sequence of the ITS region of the ribosomal RNA of LS4 was not included in the phylogenetic tree because the sequence obtained was incomplete [3]. However, the authors Obodai et al. [3] noted that the macromorphology of the LS4 sample was notably different from the other eight LS samples. As such, the reason for the marked difference in the LS4 sample can be attributed more to biotic factors than to abiotic factors. Polyunsaturated fatty acids have been reported to be dominant among the fatty acids in some edible mushrooms and could be useful for the reduction of serum cholesterol. Interestingly, similar to findings by Barros et al. [37] in which *trans* isomers of unsaturated fatty acids were not detected in various wild edible mushrooms, *trans* isomers of unsaturated fatty acids were not detected in the presently studied *Ganoderma* strains (Table 2).

Linoleic acid (C18:2n6), oleic acid (C18:1n9) and palmitic acid (C16:0) were the most abundant fatty acids present in all the *Ganoderma* strains. The same characteristic was described by other authors for *G. lucidum* from Serbia and China [31]. The percentages obtained for SFA, MUFA and PUFA in the presently studied *Ganoderma* spp. are very similar to those presented in other studies [31]. Physiological functions of linoleic acid include reduction of cardiovascular diseases, blood pressure, and arthritis as well as minimization of triglyceride levels [37,38,39].

### 2.3. Phenolic Compounds and Organic Acids

There is much interest in phenolic compounds due to their reported relation to antioxidant activity because they can act as reducing agents, free radical scavengers, single oxygen quenchers, and metal ion chelators [34,40]. Protocatechuic acid, *p*-hydroxybenzoic acid, *p*-coumaric acids and cinnamic acids were identified in strains LS2, LS6, LS8, LS9, DS1, HS1 and AM1 (Table 3). However, cinnamic acid was the only phenolic acid derivative found in strain LS4. The total phenolic composition ranged from 14 to 489 µg/100 g·dw. Strain AM1 contained the highest amounts of phenolic acids due to the high contribution of cinnamic acid. In comparison, *G. lucidum* from Portugal (1.23 mg/100 g·dw) revealed lower concentrations [40]. Nonetheless, the studied strains revealed lower contents than those of *G. lucidum* from Korea (16.2 mg/100 g·dw) [41]. There were significant differences (*p* < 0.05) observed between the strains and according to Karthikeyan et al. [42] and Korley [21], phenolics production may be influenced by the substrates and environmental conditions.

The organic acids identified in the *Ganoderma* strains included oxalic acid, malic acid and fumaric acid (Table 3). Total organic acids content of the studied *Ganoderma* spp. strains ranged between 77 and 1003 mg/100 g·dw. With the exception of strains LS8, LS9 and DS1, that presented oxalic and malic acids, and strain LS4 that only revealed oxalic acid, all the remaining strains contained all the three identified organic acids. Total organic acids content was highest in strain LS4, where oxalic acid predominated. Stojković et al. [31] chemically profiled *G. lucidum* from different origins and identified five organic acids (oxalic, quinic, malic, citric and fumaric), but revealed lower amounts.

The unique phenolic content and organic acid content of LS4 can be as a result of the genetic make-up of the samples as explained under Section 2.2.

To the best of our knowledge this is the first report revealing the organic acid content in *Ganoderma* species from Ghana.

### 2.4. Major and Trace Minerals

Mushrooms have very effective bioaccumulation mechanisms which make them take up mineral elements from the ecosystem [28]. Similar to results obtained for the nutritional composition, fatty acids and phenolic compounds in this study, the *Ganoderma* spp. showed significant (*p* < 0.05) variations in mineral elemental composition irrespective of the sample collection site as summarized in Table 4. Factors which may influence the variability of mineral elemental composition in mushrooms are both biotic and abiotic. These factors include the origin, species and/or strains and cultivation conditions of the mushrooms [43].

Phosphorus content of the *Ganoderma* strains investigated ranged between 37.7 and 472 mg/100 g·dw. The highest value was obtained for strain DS1 (472 mg/100 g·dw) which differed (*p* < 0.05) statistically from the rest of the strains investigated. Results obtained are comparable with previous studies by Ogbe and Obeka [26], who recorded values of 30.17 ppm of *G. lucidum* from Nigeria. Also, Usman et al. [24], reported values of 197.1 mg/kg from Nigeria. Since the recommended daily intake (RDI) of phosphorus is 0.7 g, *G. lucidum* is considered a good source of phosphorus [44].

Potassium content of the samples ranged between 100 and 637.9 mg/100 g·dw. Strain LS4 recorded significantly higher contents of potassium than all other strains (*p* < 0.05). Results reported by Ogbe and Obeka [26] was 1.11 ppm, while Nguyen and Park [45] reported a range of 653.8–3354.6 mg/kg. In another study by Usman et al. [24], the potassium value was 317.1 mg/kg. The RDI of potassium is 3100 mg/day [46]. Potassium, according to Duyff [47], aids in the control of blood pressure, is relevant for proper neuromuscular function, and interacts with the utilization of other minerals such as iron.

Calcium content of the samples ranged between 56.8 and 248 mg/100 g·dw. Strain DS1 recorded the highest content of calcium which differed (*p* < 0.05) from the other samples. Mhanda et al. [48] investigated minerals and trace elements in domesticated Namibian *Ganoderma* species and revealed higher levels of calcium (23.2 g/100 g of sample). Nevertheless, Nguyen and Park [45] reported 523–2398 mg calcium/kg, while Ogbe and Obeka [26] reported 1.99 ppm. Furthermore, Takshak et al. [25] recorded no values (nil) for calcium in *G. lucidum* samples collected in Haryana state in India. Calcium aids in formation of strong bones and teeth [49].

Magnesium content of the samples ranged between 43 and 204.8 mg/100 g·dw. Again strain DS1 contained a significantly higher amount of magnesium than the other samples (*p* < 0.05). Previous studies by Nguyen and Park [45] reported amounts of range 299.5–785.15 mg/kg, while Usman et al. [24] reported an amount of 8.7 g/kg, Ogbe and Obeka [26] reported 0.34 ppm.

Sodium content of the samples ranged between 0.838 and 4.53 mg/100 g·dw and recorded some significant (*p* < 0.05) differences. Values were less than published findings of Usman et al. [24] who reported 193.5 g/kg.

Sulphur content of the samples ranged between 111.2 and 187 mg/100 g·dw. Although appreciable levels of sulphur were recorded, there were significant (*p* < 0.05) differences observed in the samples. Sulphur values were comparable to values stated in literature [26,45,48].

Trace quantities of microelements were also detected (Table 4). Aluminium content of the samples ranged between 2.38 and 2.46 mg/100 g·dw and strain LS9 was observed to contain the greatest amount and differed significantly (*p* < 0.05) from all the strains. Usman et al. [24] recorded lower values of this trace element (0.2 mg/kg). Boron content ranged between <0.014 and 0.74 mg/100 g·dw and showed significant (*p* < 0.05) differences. Copper content of the samples ranged between 0.29 and 3.2 mg/100 g·dw and showed significant (*p* < 0.05) differences. Results obtained are in agreement with the ranges obtained by Mhanda et al. [48] who recorded copper content of 4.30 g/100 g, nonetheless, Ogbe and Obeka [26] reported 7.43 ppm.

Iron content of the samples ranged between 4.99–299 mg/100 g·dw and showed statistical differences (*p* < 0.05). Again, strain LS9 contained the greatest amounts. Nguyen and Park [45] reported a higher value (63.9–123.9 mg/kg). Ogbe and Obeka [26] also recorded 121.37 ppm. Takshak et al. [25] recorded an average value of 4.3 ppm. Iron is necessary for proper function of hemoglobin, and thereby the oxygen carrying capacity of red blood cells. Manganese content of the samples ranged between 0.56 and 4.22 mg/100 g·dw. Results showed statistical differences (*p* < 0.05) among samples. Manganese is important in energy production and the immune system. Also, manganese forms a complex with vitamin K to enhance blood clotting factor and with vitamin B complex to reduce effect of stress [50].

Molybdenum content of the samples ranged between <0.029 and 0.072 mg/100 g·dw for LS1–LS9 and DS1.

Zinc content of the samples ranged between 0.56 and 3.06 mg/100 g·dw. There were significant (*p* < 0.05) differences observed among the samples analyzed. Also, Ogbe and Obeka [26] reported amounts of 51.49 ppm and Mhanda et al. [48] recorded 2.89 g/100 g. Normal body development and protein synthesis are some functions of zinc in humans [50].

### 2.5. Ergosterol, Vitamin D_2_ and Tocopherols

The results obtained showed that ergosterol ranged between 113 and 373 mg/100 g·dw, while vitamin D_2_ values displayed an appreciable range from undetectable levels up to 7158 IU/100 g·dw (Table 4). Raina et al. [14] suggested that ergosterol concentrations may vary within species depending on the physiological state of the fungus. Furthermore, Stahl and Parkin [51] suggested that ergosterol concentration is strongly associated with living cytoplasmic fungi in the soil. The results obtained in this study were within ranges quantified (296–489 mg/100 g·dw) by Mattila et al. [52], who studied mushrooms cultivated in Finland. Raina et al. [14] recorded ergosterol contents of 403 µg/g for *Ganoderma lucidum*, 243 µg/g (*Calocybe indica*), 159 µg/g (*Volvariella volvacae*), and 113 µg/g (*Pleurotus florida*) from India.

The tocopherols content studied in *Ganoderma* spp. strains are presented in Table 4. Tocopherols ranged between 741 and 3191 µg/100 g·dw and showed significant (*p* < 0.05) differences regardless of the collection site. The various quantities could be related to genetic variability of the *Ganoderma* strains [43]. Some studies with wild mushrooms report the growth habitat as a very important factor influencing the profile and amounts of biomolecules with active principles [53]. According to Karthikeyan et al. [42] differences in the chemical composition of *G. lucidium* can also be associated with different collection sites. Tocopherols together with other potential antioxidants have been reported to be found in very small amounts in mushrooms [54,55], but still contribute to antioxidant properties. Plants have been reported to have higher tocopherol concentrations than mushrooms. According to Stojković et al. [56], mushrooms have tocopherol concentrations ranging between 40 and 600 μg/100 g·dw with cultivated samples possessing lower quantities than wild samples.

### 2.6. Antioxidant Activity

The antioxidant activity using the different in vitro methods is reported in Table 5. Generally, all strains of *Ganoderma* spp. revealed antioxidant properties. Strain LS4 presented the lowest antioxidant activity (highest EC_50_ values), whilst AM1 presented the highest antioxidant activity (lower EC_50_ values). High reducing power values were also recorded, using the Folin-Ciocalteu assay, for all the strains of *Ganoderma* spp. ranging between 24.3 and 147 mg gallic acid equivalents (GAE)/g of extract. These results showed statistically significant differences (*p* < 0.05) between the strains which could be explained due to the growth habitat and genetic factors. These are very important factors that could influence the profile and amounts of biomolecules with active principles [53]. These results could be correlated with the phenolic content present in the *Ganoderma* strains studied. The hydroxyl groups of phenolic compounds are responsible for the scavenging ability, contributing directly to the antioxidant action of these compounds [5].

Rajasekaran and Kalaimagal [57], recorded lower levels (42 mg GAE/g) of reducing power activity using the Folin-Ciocalteu assay in *G. lucidum* from India. In another study of *Ganoderma* spp., Stojković et al. [31] also reported lower values ranging from 1.7 and 1.3 mg GAE/g for *G. lucidum* from Serbia and China, respectively.

The reduction of Fe^3+^ into Fe^2+^ (ferricyanide/Prussian Blue assay) demonstrates the ability of antioxidants present in a sample to donate an electron, thereby stabilizing and/or terminating radical reactions. Shimada et al. [58] suggested the reducing power of medicinal mushrooms might be due to their hydrogen donating ability which is possibly related to their content of relatively high amount of reductants. The total phenolic contents of the *Ganoderma* strains presently studied were directly linked to the ferricyanide/Prussian Blue reducing activities of the strains which ranged from 0.19 to 1.07 mg/mL (Table 5).

Lipid peroxidation inhibition results ranged from 0.50 to 2.2 mg/mL for β-carotene/linoleate and 0.100–0.44 mg/mL for TBARS (Table 5). Strain LS4 presented the lowest lipid peroxidation inhibition capacity, while AM1 revealed the lowest EC_50_ values, which correspond to the best activity. Stojković et al. [31] recorded similar values (0.31 and 0.22 mg/mL) for *G. lucidum* from Serbia and China, respectively. Kim et al. [41] reported much higher EC_50_ values (10 mg/mL) for samples from Korea, than those present herein. Interestingly, *G. lucidum* from Portugal, previously studied by Heleno et al. [33], showed comparable antioxidant properties measured by the same in vitro assays.

Free radical scavenging activity of the studied species ranged between 0.38 and 4.5 g/mL for strains AM1, DS1 and LS4 respectively. Samples differed (*p* < 0.05) statistically in their radical scavenging activities. These results revealed that this medicinal mushroom is a free radical inhibitor or scavenger. The high levels of phenolic contents of the *Ganoderma* strains recorded in this present study corresponded to high levels of antioxidants.

## 3. Materials and Methods

### 3.1. Mushroom Species

Twelve wild *Ganoderma* samples were collected from the Greater Accra region of Ghana and the *Ganoderma* species strains were named and numbered as follows: collected from a *Delonix regia* tree at the University of Ghana campus, Legon, a suburb of Accra (LS1 to LS9); collected from trees at Dodowa, Haatso and Amrahia (all suburbs of Accra), strains DS1, HS1 and AM1 respectively. LS1 to LS9 samples were all matured fruiting bodies collected on 14 August 2015; DS1 was also a matured sample collected on 14 July 2015; HS1 were maturing fruiting bodies collected on 15 June 2015 and AM1 were a maturing fruiting bodies collected on 13 July 2015. Apart from LS6 and LS7 which were collected from the basal portion of the trunk of the tree, all the other LS strains were collected on various portions of the roots of the tree with a layer of soil. Based on the internal transcribed spacer region (ITS) of ribosomal DNA, the LS1 to LS9 strains have been shown to differ but belong to a single clade of a *Ganoderma lucidum* complex which clustered somewhat closely to *G. destructans* (PTA1ITS ZAF, PTA2ITS ZAF, GenBank KR183856) [3]. All samples were dried in a fabricated field dryer for 6 h and further pulverized and stored in the freezer until transported to Mountain Research Centre (CIMO), ESA, Polytechnic Institute of Bragança, Portugal, for chemical composition and antioxidant activity analysis.

### 3.2. Standards and Reagents

HPLC grade acetonitrile (99.9%) was purchased from Fisher Scientific (Lisbon, Portugal). The fatty acids methyl ester (FAME) reference standard mixture 37 (standard 47885-U), individual fatty acid isomers, sugars (d(−)-fructose, d(−)-mannitol, d(+)-raffinose pentahydrate, and d(+)-trehalose), phenolic compounds (gallic, *p*-hydroxybenzoic, *p*-coumaric, protocatechuic and cinnamic acids) and Trolox (6-hydroxy-2,5,7,8-tetramethylchroman-2-carboxylic acid) were purchased from Sigma (St. Louis, MO, USA). 2,2-Diphenyl-1-picrylhydrazyl (DPPH) was obtained from Alfa Aesar (Ward Hill, MA, USA). All other chemicals and solvents were of analytical grade and purchased from common sources. Water was treated in a Milli-Q water purification system (TGI Pure Water Systems, Greenville, SC, USA).

### 3.3. Chemical Parameters

#### 3.3.1. Nutritional and Energetic Value

Samples were analyzed for chemical composition (protein, fat, carbohydrates and ash) using AOAC procedures [59]. Crude protein content (N × 4.38) was estimated by the macro-Kjeldahl method. Crude fat was determined using a Soxhlet apparatus by extracting a known weight of sample with petroleum ether. The ash content was determined by incineration at 600 ± 15 °C. Total carbohydrates were calculated by difference and total energy was calculated according to the following equation: Energy (kcal) = 4 × (g protein + g carbohydrates) + 9 × (g fat).

#### 3.3.2. Free Sugars

The lyophilized samples (1 g) were spiked with raffinose as internal standard (IS, 5 mg/mL) and were extracted with 40 mL of 80% aqueous ethanol at 80 °C for 30 min. The resulting suspension was centrifuged at 15,000× *g* for 10 min. The supernatant was concentrated at 60 °C and defatted three times with 10 mL of ethyl ether, successively. After concentration at 40 °C, the solid residues were dissolved in water to a final volume of 5 mL and filtered through 0.2 µm nylon filters for analysis by high performance liquid chromatography coupled to a refraction index detector (HPLC-RI, Knauer, Smartline system 1000; Berlin, Germany), following previously described analysis methods [31,60]. Sugars were identified by comparing the relative retention times of sample peaks with standards. Quantification was based on the RI signal response of each standard, using raffinose as the internal standard (IS), and the results were expressed in g per 100 g of dry weight (dw).

#### 3.3.3. Beta-Glucans

Beta-glucan content was determined using a spectrophotometric kit commercially available from Megazyme (Bray, Co., Wicklow, Ireland).

#### 3.3.4. Fatty Acids

Fatty acids (obtained after Soxhlet extraction) were methylated with 5 mL of methanol:sulphuric acid 95%:toluene 2:1:1 (*v*/*v*/*v*) for, at least, 12 h in a bath at 50 °C and 160 rpm; in order to obtain phase separation deionized water (3 mL) was added; the fatty acids methyl esters (FAME) were recovered by shaking in a vortex with 3 mL of diethyl ether, and the upper phase was recouvered into an amber vial with anhydrous sodium sulphate to eliminate the water and filtered through a 0.2 µm Whatman nylon filter. Fatty acids were determined by gas-liquid chromatography (DANI model GC 1000 instrument, Milan, Italy) with flame ionization detection (GC-FID) as previously described by the authors [31,60]. Fatty acids were identified by comparing the relative retention times of FAME peaks from samples with standards. The results were recorded and processed using Clarity 4.0.1.7 Software (DataApex, Podohradska, Czech Republic) and expressed in relative percentage of each fatty acid.

#### 3.3.5. Phenolic Compounds

The lyophilized powder (1.5 g) was stirred with methanol (40 mL) at 25 °C, 150 rpm for 1 h and filtered through Whatman No. 4 paper. The residue was then extracted with an additional portion of methanol. The combined methanolic extracts were evaporated under reduced pressure (rotary evaporator Büchi R-210; Flawil, Switzerland), re-dissolved in methanol at a concentration of 5 mg/mL, filtered through 0.2 µm nylon filters and transferred into a dark injection vial. Phenolic compounds were analyzed using a Shimadzu 20A series Ultra Fast Liquid Chromatograph (UFLC, Shimadzu Corporation, Kyoto, Japan) as previously described by Stojković et al. [31]. The phenolic compounds were characterized according to their UV spectra, mass spectra and retention times compared with commercial standards when available. For quantification, a calibration curve was obtained by injection of known concentrations (5–80 µg/mL) of different standard compounds. The results were expressed in µg per 100 g of dw.

#### 3.3.6. Organic Acids

Samples (~1 g) were extracted by stirring with 25 mL of metaphosphoric acid (25 °C at 150 rpm) for 45 min and subsequently centrifuge (10 min, 4000× *g*) and filtered through a 0.2 µm nylon filter. Organic acids were measured as previously described by the authors [61]. The analysis was performed using a Shimadzu 20A series UFLC (Shimadzu) with a diode array detector (DAD), using wavelengths of 215 nm and 245 nm (for ascorbic acid). The organic acids were quantified by comparing the peak area at 215 nm with calibration curves obtained from commercial standards of each compound. The results were expressed in mg per 100 g of dw.

#### 3.3.7. Major and Trace Minerals Composition

Minerals were quantified using inductively coupled plasma (ICP) emission spectroscopy following the standard method from the American Public Health Association, Method 3120 (http://www.standardmethods.org/Store/index.cfm). Analysis was performed by the Ohio Agricultural Research and Development Center (OARDC) Star lab in Wooster, OH, USA.

#### 3.3.8. Ergosterol and Vitamin D_2_

Ergosterol and vitamin D_2_ were extracted from approximately 100 mg of dried, powdered mushrooms in a manner similar to Huang et al. [62] with some modifications. A mixture of 90:5:5 ethanol/methanol/2-propanol (4 mL) was added to the samples. Aqueous potassium hydroxide (50%) was then added to the samples, which were subsequently shaken for five hours. The resulting mixture was extracted three times with 3 mL hexane containing 0.1% butylated hydroxyl toluene assisted by probe sonication and centrifugation, pooling organic layers after each round. Aliquots (3 mL) were dried under nitrogen, resolubilized in ethanol, and filtered through a 0.2 μL nylon filter prior to analysis.

Samples were analyzed using an Agilent 1200 HPLC (Santa Clara, CA, USA) with Symmetry C18 column (4.6 × 75 mm, 3.5 μm; Waters, Milford, MA, USA). Analytes were eluted using an isocratic solvent system consisting of 92.5% methanol in water with 0.1% formic acid for 10 min followed by a wash with methyl tert-butyl ether for 2 min and a 3 min equilibration at initial conditions. The ergosterol content was screened using HPLC with photodiode array detection at 280 nm, whilst vitamin D_2_ was detected using a QTRAP 5500 mass spectrometer (AB Sciex; Framingham, MA, USA) equipped with atmospheric pressure chemical ionization operated in positive ion mode and the 397–379 amu transition was monitored.

#### 3.3.9. Tocopherols

Samples (~500 mg) were homogenized with butylated hydroxytoluene, BHT solution in hexane (10 mg/mL; 100 μL), tocol solution in hexane (IS; 50 μg/mL; 400 μL) and methanol (4 mL) by vortex mixing (1 min). Subsequently, hexane (4 mL) was added and again vortex mixed for 1 min. Saturated NaCl aqueous solution (2 mL) was added, the mixture was homogenized (1 min), centrifuged (5 min, 4000× *g*) and the clear upper layer was carefully transferred to a vial. The sample was re-extracted twice with *n*-hexane. The combined extracts were taken to dryness under a nitrogen stream, redissolved in 2 mL of *n*-hexane, dehydrated with anhydrous sodium sulphate and filtered through 0.2 µm nylon filters and transferred into a dark injection vial. Tocopherols were determined using HPLC-fluorescence detector as previously described [63]. The compounds were identified by chromatographic comparisons with authentic standards. Quantification was based on the fluorescence signal response, using the internal standard method, and tocopherols content was expressed in µg per 100 g of dw.

### 3.4. Antioxidant Activity

Five in vitro assays were used to evaluate the antioxidant activity of the samples: (1) DPPH radical scavenging activity; (2) ferricyanide/Prussian Blue; (3) Folin-Ciocalteu assay; (4) inhibition of β-carotene bleaching; and (5) thiobarbituric acid reactive substances (TBARS) as previously described by the authors [63]. Different concentrations of the extracts (previously mentioned above in Section 3.3.5.) were evaluated to find EC_50_ values (the extract concentration providing 50% of antioxidant activity calculated from the graphs of antioxidant potential against extract concentration). Trolox was used as standard.

### 3.5. Statistical Analysis

Three samples were used and all the assays were carried out in triplicate, with the exception of the vitamin D_2_ and ergosterol screening. Otherwise, results are expressed as mean values and standard deviation (SD). The results were analyzed using one-way analysis of variance (ANOVA) followed by Tukey’s HSD Test with *p* = 0.05. Analysis was carried out using SPSS v. 23.0 (IBM Corp., Armonk, NY, USA).

## 4. Conclusions

The chemical and bioactive properties of *G. lucidum* proved to be highly dependent on the strains of the samples (biotic factors) than on the collection site (abiotic factors). The *Ganoderma* species revealed important nutrient and bioactive molecules such as reducing sugars, organic acids, phenolic compounds, unsaturated and saturated fatty acids, tocopherols, ergosterol, vitamin D and β-glucans. Further research on the production of these species and the extraction of bioactive metabolites will inform the development of efficient biotechnological methods to grow as well as obtain these metabolites.

## Figures and Tables

**Table 1:** Nutritional value, sugars and beta-glucan composition of the studied wild mushrooms.

Sample	Nutritional Value					Composition in Sugars						Beta-Glucan (µg/g·dw)
	Fat	Protein	Ash	Carbohydrates	Energetic Value	Ramnose	Fructose	Mannitol	Sucrose	Trehalose	Total Sugars	
LS1	0.78 ± 0.01 f	24.5 ± 0.3 a	1.4 ± 0.2 c,d	73.31 ± 0.08 g	398.4 ± 0.5 d	1.63 ± 0.04 b	1.39 ± 0.05 b	1.62 ± 0.02 b	2.85 ± 0.01 b	1.41 ± 0.01 b	8.9 ± 0.1 b	0.98 ± 0.02 f
LS2	0.95 ± 0.05 d	23 ± 1 b,c	0.9 ± 0.1 f	75.4 ± 0.7 d,e	401.2 ± 0.2 c	1.20 ± 0.01 d	0.42 ± 0.01 d	0.71 ± 0.04 e	2.03 ± 0.01 d	0.77 ± 0.03 c	5.13 ± 0.05 d	2.45 ± 0.07 b
LS3	0.78 ± 0.01 f	22.5 ± 0.3 c	1.5 ± 0.1 b,c	75.2 ± 0.2 d,e,f	398.0 ± 0.3 d	0.58 ± 0.01 f	0.123 ± 0.005 e	0.28 ± 0.01 h	1.00 ± 0.03 g	0.38 ± 0.01 e	2.37 ± 0.03 f	1.12 ± 0.03 d
LS4	0.87 ± 0.01 e	22.6 ± 0.1 c	1.47 ± 0.05 b,c	75.08 ± 0.07 d,e,f	398.4 ± 0.1 d	0.56 ± 0.01 f	nd	nd	1.19 ± 0.06 f	nd	1.75 ± 0.06 h	0.80 ± 0.01 g
LS5	0.91 ± 0.02 d,e	24 ± 1 a	0.68 ± 0.01 g	74.3 ± 0.8 f,g	401.84 ± 0.07 b,c	1.49 ± 0.07 c	1.16 ± 0.01 c	1.24 ± 0.08 c	2.61 ± 0.06 c	1.58 ± 0.07 a	8.1 ± 0.1 c	2.67 ± 0.04 b
LS6	1.16 ± 0.06 c	22.7 ± 0.1 b,c	0.9 ± 0.1 f	75.21 ± 0.07 d,e,f	402.2 ± 0.5 a,b	0.87 ± 0.04 e	0.41 ± 0.04 d	0.57 ± 0.02 f	1.50 ± 0.08 e	0.62 ± 0.03 d	3.97 ± 0.09 e	1.83 ± 0.01 c
LS7	0.53 ± 0.03 g	23.7 ± 0.2 a,b	1.19 ± 0.06 d,e	74.6 ± 0.2 e,f	397.9 ± 0.3 d	2.76 ± 0.09 a	3.05 ± 0.07 a	2.09 ± 0.03 a	4.78 ± 0.08 a	1.37 ± 0.04 b	14.1 ± 0.1 a	11.47 ± 0.03 a
LS8	1.31 ± 0.05 b	22.2 ± 0.3 c	1.18 ± 0.08 d,e	75.3 ± 0.1 d,e	401.85 ± 0.05 b,c	0.43 ± 0.02 g	0.063 ± 0.001 f	0.15 ± 0.01 i	0.69 ± 0.02 h	0.065 ± 0.003 g	1.39 ± 0.03 i	2.49 ± 0.08 b
LS9	1.11 ± 0.03 c	22.2 ± 0.3 c	0.88 ± 0.06 f	75.9 ± 0.3 d	402.02 ± 0.06 b,c	0.29 ± 0.02 h	0.041 ± 0.001 f	0.16 ± 0.01 i	0.44 ± 0.03 i	0.13 ± 0.01 f	1.06 ± 0.05 j	1.06 ± 0.06 e
DS1	0.94 ± 0.05 d	18.4 ± 0.1 e	2.12 ± 0.08 a	78.55 ± 0.01 b	396.22 ± 0.03 e	0.42 ± 0.03 g	0.044 ± 0.003 f	0.82 ± 0.03 d	0.72 ± 0.02 h	0.18 ± 0.04 f	2.2 ± 0.1 g	tr
HS1	0.48 ± 0.01 g	20.4 ± 0.4 d	1.57 ± 0.08 b	77.54 ± 0.21 c	396.1 ± 0.2 e	0.104 ± 0.001 i	0.024 ± 0.002 f	0.063 ± 0.002 j	0.21 ± 0.01 j	0.061 ± 0.004 g	0.457 ± 0.006 l	nd
AM1	1.40 ± 0.02 a	15.7 ± 0.1 f	1.0 ± 0.1 e,f	81.90 ± 0.01 a	402.9 ± 0.5 a	0.155 ± 0.001 i	0.045 ± 0.002 f	0.43 ± 0.02 g	0.249 ± 0.001 j	nd	0.88 ± 0.02 k	nd

In each column different letters (“a” to “l”, being “a” the highest value) mean statistically significant differences between samples (*p* < 0.05). nd—not detected; tr—traces. With the exception of energetic value reported in Kcal/100 g·dw, the nutritional values and composition in sugars are recorded in g/100 g·dw.

**Table 2:** Composition of fatty acids of the studied wild mushrooms.

	LS1	LS2	LS3	LS4	LS5	LS6	LS7	LS8	LS9	DS1	HS1	AM1
C6:0	0.29 ± 0.01	0.108 ± 0.006	0.059 ± 0.001	0.160 ± 0.004	0.161 ± 0.004	0.132 ± 0.004	0.311 ± 0.002	0.111 ± 0.001	0.054 ± 0.001	0.110 ± 0.001	0.058 ± 0.002	0.110 ± 0.006
C8:0	0.44 ± 0.03	0.17 ± 0.01	0.115 ± 0.004	0.51 ± 0.01	0.51 ± 0.01	0.140 ± 0.008	0.222 ± 0.003	0.30 ± 0.02	0.164 ± 0.001	0.35 ± 0.01	0.34 ± 0.01	0.19 ± 0.01
C10:0	0.57 ± 0.02	0.232 ± 0.007	0.146 ± 0.005	0.36 ± 0.01	0.36 ± 0.01	0.224 ± 0.003	0.36 ± 0.02	0.34 ± 0.02	0.180 ± 0.004	0.32 ± 0.03	0.28 ± 0.02	0.22 ± 0.01
C12:0	0.57 ± 0.01	0.30 ± 0.01	0.24 ± 0.01	0.57 ± 0.03	0.58 ± 0.03	0.343 ± 0.004	0.48 ± 0.02	0.45 ± 0.03	0.262 ± 0.004	0.56 ± 0.06	0.556 ± 0.002	0.26 ± 0.01
C14:0	1.05 ± 0.01	0.62 ± 0.03	0.60 ± 0.01	1.16 ± 0.04	1.16 ± 0.04	0.73 ± 0.01	0.96 ± 0.02	0.90 ± 0.02	0.56 ± 0.01	0.99 ± 0.07	1.11 ± 0.02	0.59 ± 0.02
C14:1	0.52 ± 0.02	0.60 ± 0.01	0.354 ± 0.002	1.21 ± 0.02	1.22 ± 0.02	0.010 ± 0.001	0.019 ± 0.001	0.015 ± 0.001	0.010 ± 0.001	0.173 ± 0.004	0.34 ± 0.03	0.059 ± 0.004
C15:0	1.03 ± 0.01	0.95 ± 0.01	0.72 ± 0.01	0.79 ± 0.01	0.79 ± 0.01	0.69 ± 0.01	0.93 ± 0.02	0.44 ± 0.01	0.376 ± 0.004	0.73 ± 0.06	1.03 ± 0.01	1.08 ± 0.01
C16:0	14.65 ± 0.08	15.69 ± 0.12	21.73 ± 0.06	26.80 ± 0.45	26.96 ± 0.41	21.52 ± 0.11	16.37 ± 0.19	23.76 ± 0.11	23.72 ± 0.09	19.08 ± 0.12	23.55 ± 0.06	15.89 ± 0.08
C16:1	0.28 ± 0.01	0.30 ± 0.01	0.547 ± 0.004	0.82 ± 0.01	0.82 ± 0.01	0.64 ± 0.01	0.32 ± 0.01	1.10 ± 0.01	1.15 ± 0.02	0.301 ± 0.004	0.419 ± 0.005	0.437 ± 0.002
C17:0	1.13 ± 0.01	1.19 ± 0.07	1.49 ± 0.01	1.66 ± 0.01	1.67 ± 0.02	1.46 ± 0.01	1.16 ± 0.01	1.32 ± 0.01	1.18 ± 0.01	1.34 ± 0.07	2.63 ± 0.01	1.03 ± 0.01
C18:0	5.68 ± 0.05	6.09 ± 0.03	7.63 ± 0.03	12.27 ± 0.04	12.35 ± 0.06	8.58 ± 0.02	6.39 ± 0.04	8.89 ± 0.03	8.56 ± 0.01	9.91 ± 0.33	16.03 ± 0.09	5.70 ± 0.02
C18:1n9	21.78 ± 0.07	25.97 ± 0.10	39.44 ± 0.02	35.03 ± 0.09	35.25 ± 0.02	42.28 ± 0.03	26.19 ± 0.10	45.80 ± 0.15	48.76 ± 0.14	24.82 ± 0.29	22.55 ± 0.14	28.81 ± 0.04
C18:2n6	44.27 ± 0.09	40.09 ± 0.09	21.83 ± 0.01	9.74 ± 0.12	9.80 ± 0.14	17.53 ± 0.01	40.96 ± 0.04	7.57 ± 0.01	7.48 ± 0.02	30.55 ± 0.23	17.12 ± 0.08	40.43 ± 0.12
C18:3n3	0.247 ± 0.004	0.23 ± 0.01	0.153 ± 0.005	0.167 ± 0.007	0.168 ± 0.007	0.140 ± 0.001	0.30 ± 0.02	0.210 ± 0.004	0.43 ± 0.02	0.33 ± 0.02	0.491 ± 0.003	0.34 ± 0.02
C20:0	0.44 ± 0.02	0.44 ± 0.02	0.69 ± 0.04	1.14 ± 0.05	1.15 ± 0.04	0.77 ± 0.01	0.44 ± 0.01	0.799 ± 0.001	0.86 ± 0.01	0.77 ± 0.01	1.14 ± 0.10	0.36 ± 0.03
C20:1	0.095 ± 0.001	0.117 ± 0.008	0.081 ± 0.006	0.131 ± 0.001	0.132 ± 0.001	0.082 ± 0.004	0.123 ± 0.006	0.086 ± 0.001	0.055 ± 0.001	0.053 ± 0.004	nd	0.120 ± 0.001
C20:2	0.057 ± 0.004	0.051 ± 0.004	0.061 ± 0.001	0.076 ± 0.006	0.076 ± 0.006	0.073 ± 0.006	0.012 ± 0.002	0.064 ± 0.002	0.056 ± 0.001	0.060 ± 0.001	nd	0.038 ± 0.002
C20:3	0.26 ± 0.01	0.35 ± 0.03	0.215 ± 0.001	0.48 ± 0.05	0.48 ± 0.05	0.177 ± 0.001	0.29 ± 0.02	0.118 ± 0.002	0.090 ± 0.001	0.38 ± 0.02	0.40 ± 0.01	0.23 ± 0.02
C20:5n3	0.27 ± 0.01	0.129 ± 0.001	0.227 ± 0.002	0.36 ± 0.01	0.37 ± 0.01	nd	0.206 ± 0.003	0.353 ± 0.001	0.35 ± 0.01	0.142 ± 0.001	0.99 ± 0.01	0.18 ± 0.01
C22:0	1.58 ± 0.05	1.76 ± 0.14	1.29 ± 0.03	3.09 ± 0.20	2.50 ± 0.02	1.20 ± 0.06	1.34 ± 0.02	1.27 ± 0.01	0.99 ± 0.05	1.55 ± 0.03	2.26 ± 0.06	1.16 ± 0.01
C22:1n9	0.35 ± 0.01	0.34 ± 0.04	0.118 ± 0.002	0.44 ± 0.04	0.45 ± 0.04	0.22 ± 0.01	0.35 ± 0.03	0.27 ± 0.01	0.282 ± 0.003	0.66 ± 0.06	0.72 ± 0.02	0.21 ± 0.01
C23:0	0.72 ± 0.02	1.07 ± 0.02	0.773 ± 0.002	1.19 ± 0.07	1.20 ± 0.07	0.58 ± 0.01	0.52 ± 0.01	0.93 ± 0.01	1.03 ± 0.01	1.07 ± 0.01	1.79 ± 0.04	0.87 ± 0.01
C24:0	3.73 ± 0.27	3.22 ± 0.11	1.50 ± 0.02	1.85 ± 0.18	1.86 ± 0.19	2.49 ± 0.06	1.78 ± 0.05	4.91 ± 0.03	3.40 ± 0.07	5.75 ± 0.06	6.20 ± 0.10	1.67 ± 0.02
Total SFA (% of total FA)	31.9 ± 0.2 h	31.8 ± 0.2 h	36.98 ± 0.01 g	51.6 ± 0.1 b	51.25 ± 0.19 b	38.9 ± 0.1 f	31.3 ± 0.2 i	44.4 ± 0.1 c	41.3 ± 0.1 e	42.5 ± 0.6 d	57 ± 1 a	29.1 ± 0.1 j
Total MUFA (% of total FA)	23.0 ± 0.1 j	27.3 ± 0.1 g	40.54 ± 0.01 d	37.6 ± 0.1 e	37.87 ± 0.01 e	43.2 ± 0.1 c	27.0 ± 0.1 g	47.3 ± 0.2 b	50.3 ± 0.1 a	26.0 ± 0.3 h	24.0 ± 0.1 i	29.64 ± 0.02 f
Total PUFA (% of total FA)	45.1 ± 0.1 a	40.9 ± 0.1 d	22.49 ± 0.01 f	10.8 ± 0.2 i	10.88 ± 0.20 i	17.9 ± 0.1 h	41.8 ± 0.1 b	8.31 ± 0.01 j	8.41 ± 0.01 j	31.5 ± 0.3 e	19.0 ± 0.1 g	41.2 ± 0.1 c

nd—not detected; SFA—Saturated fatty acids; MUFA—Monounsaturated fatty acids; PUFA—Polyunsaturated fatty acids. In the total SFA, MUFA and PUFA rows different letters (“a” to “j”, being “a” the highest value) mean statistically significant differences between samples (*p* < 0.05). nd—not detected; SFA—Saturated fatty acids; MUFA—Monounsaturated fatty acids; PUFA—Polyunsaturated fatty acids. In the total SFA, MUFA and PUFA rows different letters (“a” to “j”, being “a” the highest value) mean statistically significant differences between samples (*p* < 0.05).

**Table 3:** Composition in phenolic compounds (µg/100 g·dw) and organic acids (mg/100 g·dw) of the studied wild mushrooms.

Sample	Phenolic Compounds					Other Organic Compounds			
	Protocatechuic Acid	*p*-Hydroxybenzoic Acid	*p*-Coumaric Acid	Total Phenolic Compounds	Cinamic Acid	Oxalic Acid	Malic Acid	Fumaric Acid	Total Organic Compounds
LS1	14 ± 2 f	nd	nd	14 ± 2 j	15.8 ± 0.2 e,f	111 ± 1 f	88.29 ± 0.04 a	8.4 ± 0.2 a	208 ± 2 d
LS2	65.6 ± 0.1 b	57.9 ± 0.2 f	9.4 ± 0.1 d	132.9 ± 0.2 e	19 ± 4 d,e	125.3 ± 0.3 d	64 ± 1 e	7.92 ± 0.01 a	198 ± 1 e
LS3	7.6 ± 0.2 g	nd	nd	7.6 ± 0.2 k	8.3 ± 0.2 g	546 ± 1 b	39 ± 1 g	0.13 ± 0.02 c	586 ± 1 b
LS4	nd	nd	nd	-	14 ± 2 f	1003 ± 1 a	nd	nd	1003 ± 1 a
LS5	13.7 ± 0.3 f	22.7 ± 0.1 h	nd	36.4 ± 0.4 i	12.7 ± 0.4 f	145 ± 1 c	81 ± 3 b	3.06 ± 0.05 b	229 ± 2 c
LS6	87 ± 1 a	126 ± 4 c	3.8 ± 0.1 g	217 ± 5 c	50.4 ± 0.4 b	85 ± 1 h	86.6 ± 0.2 a	0.5 ± 0.1 c	172 ± 1 h
LS7	nd	107 ± 1 d	nd	107 ± 1 f	51 ± 4 b	101.4 ± 0.5 g	73 ± 1 c	8 ± 1 a	183 ± 2 g
LS8	55 ± 2 d	126 ± 3 c	19.9 ± 0.2 b	201 ± 1 d	48.7 ± 0.9 b	112.3 ± 0.4 f	38 ± 1 g	nd	150 ± 1 i
LS9	50.8 ± 0.2 e	34.7 ± 0.3 g	15.1 ± 0.2 c	101 ± 1 g	21.5 ± 0.3 d	119 ± 1 e	67 ± 1 d	nd	186.1 ± 0.4 f
DS1	59 ± 1 c	176 ± 1 b	7.0 ± 0.1 e	242 ± 1 b	39.5 ± 0.6 c	79 ± 2 i	57 ± 1 f	nd	135.8 ± 0.5 j
HS1	16.6 ± 0.3 f	67 ± 1 e	6.5 ± 0.1 f	90 ± 1 h	1.2 ± 0.1 h	52.2 ± 0.1 j	22 ± 1 h	3.61 ± 0.01 b	78 ± 1 k
AM1	85 ± 4 a	331 ± 7 a	73 ± 1 a	489 ± 4 a	234.8 ± 1.9 a	51.7 ± 0.1 j	22 ± 1 h	3.57 ± 0.01 b	77 ± 1 k

In each column different letters (“a” to “k”, being “a” the highest value) mean statistically significant differences between samples (*p* < 0.05). nd—not detected.

**Table 4:** Mineral Composition (major & trace) (mg/100 g·dw), ergosterol (mg/100 g·dw), vitamin D_2_ (IU/100 g·dw) and tocopherol (µg/100 g·dw) contents of studied edible mushrooms.

Component		LS1	LS2	LS3	LS4	LS5	LS6	LS7	LS8	LS9	DS1	HS1	AM1
Major mineral elements	P	167 ± 1 d	193.6 ± 0.8 b	106.1 ± 0.3 f	186 ± 1 c	102.4 ± 0.6 g	37.7 ± 0.1 j	129.0 ± 0.2 e	49.1 ± 0.1 i	79.7 ± 0.1 h	472 ± 2 a	nd	nd
	K	438.6 ± 0.4 d	471.4 ± 0.9 b	371 ± 1 f	637.9 ± 0.2 a	273.2 ± 0.5 g	226.9 ± 0.2 h	429.5 ± 0.7 e	100 ± 2 j	187 ± 1 i	452.4 ± 0.7 c	nd	nd
	Ca	83.0 ± 0.4 f	141.6 ± 0.5 c	75.2 ± 0.2 h	80 ± 1 g	83.6 ± 0.3 f	124 ± 1 d	56.8 ± 0.7 i	96.4 ± 0.4 e	147.8 ± 0.3 b	248 ± 1 a	nd	nd
	Mg	56.1 ± 1.4 g	71.5 ± 0.1 e	66.4 ± 0.2 f	73.9 ± 0.3 d	55 ± 1 g	102.9 ± 0.8 b	48 ± 1 h	43 ± 1 i	93 ± 1 c	204.8 ± 0.2 a	nd	nd
	Na	0.838 ± 0.003 h	0.89 ± 0.03 h	0.913 ± 0.004 h	3.43 ± 0.04 d	1.03 ± 0.03 g	3.7 ± 0.1 c	1.57 ± 0.01 f	2.57 ± 0.06 e	4.37 ± 0.04 b	4.53 ± 0.01 a	nd	nd
	S	147 ± 3 d	165.6 ± 0.2 b	155 ± 2 c	169 ± 1 b	144 ± 1 de	119 ± 2 g	128 ± 4 f	140 ± 3 e	111.2 ± 0.4 h	187 ± 2 a	nd	nd
Trace elements	Al	7.6 ± 0.1 e,f	3.07 ± 0.03 g,h	35.80 ± 0.04 c	9.30 ± 0.04 e	4.80 ± 0.07 f,g,h	67 ± 4 b	2.38 ± 0.03 h	26.9 ± 0.4 d	246 ± 4 a	7.0 ± 0.1 ef	nd	nd
	B	0.49 ± 0.01 c	0.49 ± 0.03 c	<0.014 e	<0.014 e	0.54 ± 0.01 b	0.23 ± 0.01 d	0.74 ± 0.01 a	0.26 ± 0.01 d	<0.014 e	0.52 ± 0.03 bc	nd	nd
	Cu	0.68 ± 0.01 c,d	0.60 ± 0.02 d	0.49 ± 0.04 e	1.05 ± 0.01 b	0.45 ± 0.05 e	0.44 ± 0.01 e	0.73 ± 0.06 c	0.29 ± 0.02 f	0.34 ± 0.04 f	3.2 ± 0.1 a	nd	nd
	Fe	10.5 ± 0.1 e,f	8.2 ± 0.2 f	29.1 ± 0.1 d	10.9 ± 0.4 e	8.6 ± 0.5 ef	57.64 ± 3.03 b	4.99 ± 0.09 g	35.1 ± 0.2 c	299 ± 2 a	8.88 ± 0.06 ef	nd	nd
	Mn	2.0 ± 0.2 b	1.58 ± 0.08 d,e	1.86 ± 0.03 bc	1.83 ± 0.06 c	1.73 ± 0.03 cd	1.43 ± 0.06 e	0.56 ± 0.03 g	1.46 ± 0.07 e	4.22 ± 0.06 a	1.12 ± 0.01 f	nd	nd
	Mo	<0.029	<0.029	<0.029	<0.029	<0.029	<0.029	<0.029	<0.029	<0.029	0.072 ± 0.001	nd	nd
	Zn	1.64 ± 0.04 c	2.0 ± 0.1 b	1.61 ± 0.08 c	3.06 ± 0.01 a	1.14 ± 0.01 e	0.71 ± 0.01 f	1.46 ± 0.04 d	0.56 ± 0.04 g	1.18 ± 0.01 e	1.38 ± 0.03 d	nd	nd
Vitamins	Ergosterol	296 ± 6 d	373 ± 2 a	310 ± 6 c	288 ± 3 d	328 ± 1 b	113 ± 4 g	308 ± 1 c	237 ± 3 e	331 ± 3 b	151 ± 6 f	nd	nd
	Vitamin D_2_	4692 ± 57 d	4338 ± 42 e	3389 ± 14 g	6636 ± 17 b	7158 ± 19 a	891 ± 18 i	4904 ± 13 c	3660 ± 21 f	3129 ± 14 h	nd	nd	nd
	α-tocopherol	22 ± 1 c	22 ± 1 c	19 ± 1 d	nd	55 ± 0.5 a	38 ± 1 b	16 ± 2 d	17 ± 1 d	18 ± 2 d	53 ± 3 a	18 ± 1 d	9.45 ± 0.02 e
	β-tocopherol	719 ± 1 l	1730 ± 2 e	2653 ± 1 b	2592 ± 1 c	3136 ± 12 a	1860 ± 2 d	1137 ± 2 j	1360 ± 2 h	1660 ± 3 f	1295 ± 1 i	1369 ± 2 g	796 ± 4 k
	Total tocopherols	741 ± 2 k	1752 ± 3 e	2671 ± 1 b	2592 ± 1 c	3191 ± 12 a	1898 ± 3 d	1153 ± 3 i	1378 ± 3 g	1678 ± 5 f	1349 ± 2 h	1386 ± 3 g	806 ± 4 j

nd—not detected. In each row different letters (“a” to “l”, being “a” the highest value) mean statistically significant differences between samples (*p* < 0.05).

**Table 5:** Antioxidant activity of the studied edible mushrooms.

Sample	Reducing Power		Radical Scavenging Activity	Lipid Peroxidation Inhibition	
	Folin-Ciocalteu (mg GAE/g Extract)	Ferricyanide/Prussian Blue (EC_50_; mg/mL)	DPPH Scavenging Activity (EC_50_; mg/mL)	β-Carotene/Linoleate (EC_50_; mg/mL)	TBARS (EC_50_; mg/mL)
LS1	53.5 ± 0.4 i	0.85 ± 0.01 c	2.26 ± 0.03 c	1.5 ± 0.1 c	0.36 ± 0.01 b
LS2	79 ± 1 e	0.34 ± 0.01 g	1.52 ± 0.04 e,f	1.00 ± 0.05 e,f	0.24 ± 0.03 e
LS3	35.4 ± 0.1 j	0.96 ± 0.01 b	4.1 ± 0.2 b	1.9 ± 0.1 b	0.36 ± 0.04 b
LS4	24.3 ± 0.2 k	1.07 ± 0.01 a	4.5 ± 0.1 a	2.2 ± 0.1 a	0.44 ± 0.01 a
LS5	63.7 ± 0.6 h	0.67 ± 0.01 d	1.7 ± 0.1 d	1.33 ± 0.02 c,d	0.28 ± 0.01 c
LS6	101 ± 1 c	0.24 ± 0.01 i	1.02 ± 0.04 g	0.90 ± 0.04 f	0.22 ± 0.01 e,f
LS7	75 ± 2 f	0.43 ± 0.01 f	1.56 ± 0.02 e	1.04 ± 0.05 e	0.24 ± 0.03 e
LS8	86 ± 1 d	0.27 ± 0.01 h	1.42 ± 0.01 f	0.9 ± 0.1 e,f	0.22 ± 0.03 d
LS9	70 ± 1 g	0.44 ± 0.01 f	1.57 ± 0.04 e	1.23 ± 0.02 d	0.26 ± 0.03 c,d
DS1	124 ± 1 b	0.23 ± 0.01 i	0.38 ± 0.01 h	0.90 ± 0.04 f	0.19 ± 0.01 f
HS1	70 ± 1 g	0.64 ± 0.02 e	1.59 ± 0.04 e	1.3 ± 0.2 d	0.35 ± 0.03 b
AM1	147 ± 2 a	0.19 ± 0.01 j	0.38 ± 0.01 h	0.50 ± 0.03 g	0.100 ± 0.002 g

Results of antioxidant activity are expressed in EC_50_ values: sample concentration providing 50% of antioxidant activity or 0.5 of absorbance in the reducing power. In each column different letters (“a” to “k”, being “a” the highest value) mean statistically significant differences between samples (*p* < 0.05).
